# Clinical management of hypophosphatemic osteomalacia induced by adefovir and tenofovir: Insights from a case report

**DOI:** 10.1097/MD.0000000000040746

**Published:** 2024-11-29

**Authors:** Yinhui He, Xin Huang, Yongjun Ye, Haiyan Xu

**Affiliations:** a Department of Endocrinology, Lishui Central Hospital, the Fifth Affiliated Hospital of Wenzhou Medical University, Lishui, Zhejiang Province, China; b Department of Radiology, Lishui Central Hospital, the Fifth Affiliated Hospital of Wenzhou Medical University, Lishui, Zhejiang Province, China.

**Keywords:** adefovir dipivoxil, chronic viral hepatitis B, hypophosphatemic rickets, osteomalacia, tenofovir disoproxil fumarate

## Abstract

**Rationale:**

Hypophosphatemic osteomalacia is a rare chronic metabolic bone disease characterized by low serum phosphate levels owing to genetic or acquired causes. This article presents a case report of the clinical management, challenges encountered, and prognosis of secondary hypophosphatemic osteomalacia induced by defovir and tenofovir.

**Patient concerns:**

A 55-year-old male patient had been experiencing persistent dull chest pain and fatigue for more than a year. The patient had chronic hepatitis B infection for over 10 years, with regular use of adefovir dipivoxil capsules for more than 10 years. Five months before admission, the patient was switched to tenofovir alafenamide fumarate tablets.

**Diagnoses:**

After obtaining clinical manifestations, medical history, and examination results, tumor-induced osteomalacia was excluded, and the final diagnosis was drug-induced hypophosphatemic osteomalacia.

**Interventions:**

Adefovir dipivoxil and tenofovir alafenamide were discontinued, and the patient was switched to entecavir disintegration tablets for antiviral therapy. He was advised to follow a high-phosphate diet, receive phosphorus supplementation and calcitriol capsules to promote calcium absorption, obtain moderate sun exposure, and take measures to prevent falls and fractures.

**Outcomes:**

Serum phosphate levels showed a gradual upward trend, with the most recent measurement being 0.85 mmol/L. The bone density gradually improved and reached normal levels in the most recent assessment. The symptoms of fatigue and chest pain were resolved.

**Lessons:**

Accurate diagnosis requires a combination of clinical presentation, medical history, biochemical and radiological findings, and, if available, measurement of fibroblast growth factor 23 (FGF 23). The role of national, provincial, or regional centers for rare diseases is crucial for conducting unconventional tests and providing access to rare medications.

## 1. Introduction

Hypophosphatemic rickets/osteomalacia is a group of rare chronic metabolic bone diseases characterized by low blood phosphate levels owing to genetic or acquired causes. In adults, it is known as osteomalacia and presents with fatigue, bone pain, multiple fractures, and limited mobility.^[1]^ Drug-induced renal tubular damage, or Fanconi syndrome, is a common cause of acquired hypophosphatemic osteomalacia. Most cases of drug-induced hypophosphatemic osteomalacia are associated with a single medication.^[2–4]^ This article reports a case of hypophosphatemic osteomalacia caused by sequential treatment with adefovir dipivoxil and tenofovir alafenamide, and summarizes its clinical management.

## 2. Case summary

**Figure 1.**
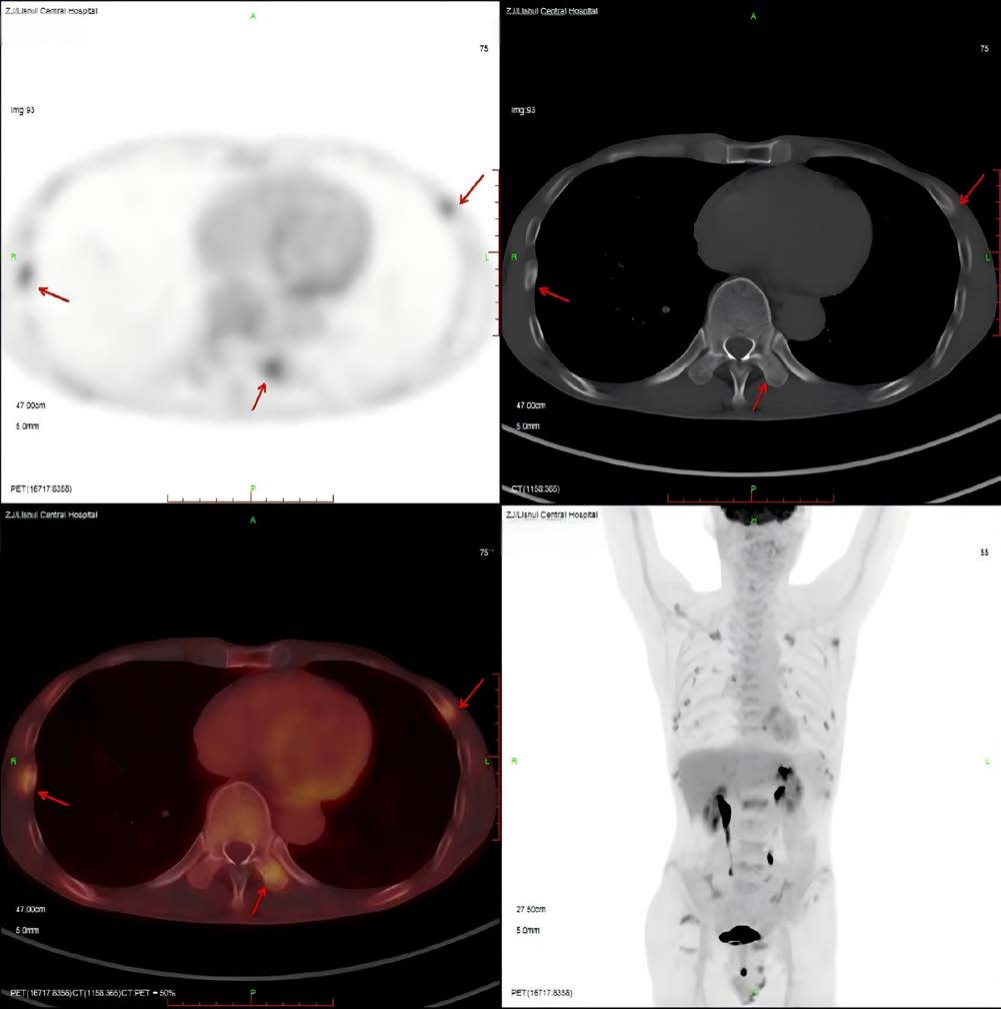
Positron emission tomography-computed tomography.

A 55-year-old male patient of Han ethnicity with junior high school education and a farmer by occupation was admitted to our department on November 9, 2020, due to “chest pain for over a year.” The patient had experienced persistent dull chest pain for over a year, which did not worsen with movement. He also reported fatigue and no abdominal pain or diarrhea. He had previously sought medical attention at a local county hospital, where a CT scan revealed multiple fractures of the right ribs 3 to 10 and left ribs 2 to 8 with callus formation. No medication was prescribed, and the patient was advised to rest at home. The patient’s chest pain did not improve significantly over the next 7 months, and he sought medical attention at another hospital in the county town. Blood tests showed a phosphate level of 0.41 mmol/L, and osteoporosis was suspected. The patient received symptomatic supportive treatment with alendronate sodium, calcium phosphate D3 chewable tablets, and alfacalcidol capsules. Two follow-up bone density scans during treatment showed poor treatment response, indicating osteoporosis. Subsequently, he visited our hospital and an outpatient visit revealed osteoporosis based on dual-energy X-ray absorptiometry. Whole-body positron emission tomography-computed tomography (PET-CT) showed increased FDG metabolism in multiple bones with fractures (Fig. 1). The patient did not have chills, fever, or chest tightness but was admitted to our hospital with a provisional diagnosis of hypophosphatemic osteomalacia. During hospitalization, the patient had clear consciousness, normal mental state, poor appetite, disturbed sleep, normal bowel movements, and weight loss of approximately 5 kg. His medical history included chronic hepatitis B virus (HBV) infection for over 10 years and regular use of adefovir dipivoxil capsules for more than 10 years. Five months before admission, the patient was switched to tenofovir alafenamide fumarate tablets. His marital and reproductive histories were unremarkable, and his family history was significant for his father’s death from lung cancer. The patient’s mother and siblings were healthy. Upon admission, vital signs were normal and tenderness was observed on the chest wall. Further investigation revealed that the results are detailed in Table 1. The patient tested positive for hepatitis B surface antigen, hepatitis B e antibody, and hepatitis B core antibody, but negative for HBV-DNA. There were no significant abnormalities in tumor markers, specific protein myeloma series, adrenocorticotropic hormone, growth hormone, cortisol, thyroid function, sex hormones, or the aldosterone/renin ratio. Urine analysis revealed microalbuminuria, elevated β2-microglobulin, and elevated α1-microglobulin levels. Blood gas analysis revealed metabolic acidosis. Repeat tests showed elevated urinary calcium and phosphorus levels as well as 24-hour urinary calcium and phosphorus excretion. Repeated blood tests revealed elevated creatinine, normal uric acid, decreased estimated glomerular filtration rate, decreased chloride, decreased calcium, and decreased phosphate levels. CT tomography showed thickening of both maxillary sinus mucosae, which was more prominent on the right side, with fluid accumulation on the left side, suggesting inflammatory changes. Whole-body bone scintigraphy showed abnormally increased bone metabolism and shallow renal parenchymal uptake.

**Table 1:** Biochemical results of serum and urinary index.

Serum AND urine biochemical indicators	Results	Normal reference values
Serum phosphorus (mmol/L)	0.30	0.85 to 1.51
Serum calcium (mmol/L)	2.19	2.11 to 2.52
Parathyroid hormone (pg/mL)	97.6	15 to 65
Serum creatinine (μmol/L)	148	57 to 97
Fasting blood glucose (mmol/L)	3.97	3.9 to 6.1
Serum uric acid (μmol/L)	94	208 to 428
Alkaline phosphatase (U/L)	161	45 to 125
25-hydroxy vitamin D (ng/mL)	22.7	>20
β-Collagen special sequence (pg/mL)	564	43 to 783
N-MID osteocalcin (ng/mL)	16.65	6.0 to 24.66
Total type I collagen amino-terminal propeptide (ng/mL)	47.76	9.06 to 76.24
Urinary pH	6.5	4.5 to 8.0
Urinary glucose	3+	Negative
Urinary protein	3+	Negative
24-h urinary protein excretion (g/24 h)	1.22	<0.15
Urinary phosphorus (mmol/L)	5.71	0.85 to 1.51
Urinary calcium (mmol/L)	4.6	/
24-h urinary calcium (mmol/24 h)	5.52	1.0 to 7.5
24-h urinary phosphorus (mmol/24 h)	6.85	12 to 42

Final diagnosis: drug-induced hypophosphatemic osteomalacia, Fanconi syndrome, chronic hepatitis B virus, and Renal insufficiency.

### 2.1. Treatment course

**Figure 2.**
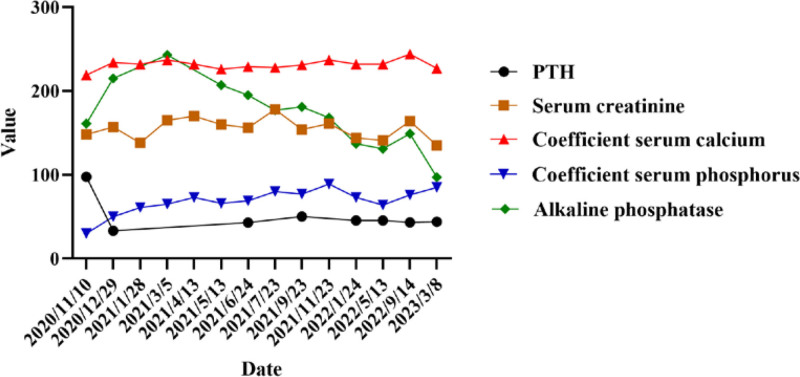
Follow-up data of the patient.

Adefovir dipivoxil and tenofovir alafenamide were discontinued, and the patient was switched to entecavir disintegration tablets for antiviral therapy. He was advised to follow a high-phosphate diet, obtain moderate sun exposure, and take measures to prevent falls and fractures. During hospitalization, the patient received calcium carbonate tablets for calcium supplementation, calcitriol capsules to promote calcium absorption, and sodium glycerophosphate injections for phosphorus supplementation. After discharge, the patient was unable to obtain a neutral phosphate oral solution and received phosphate supplementation with sodium glycerophosphate. Calcium carbonate tablets were discontinued in the later stages. The patient was followed-up multiple times at our hospital. To facilitate graphing, the coefficient of serum calcium was calculated as the measured serum calcium multiplied by 100, and the coefficient of serum phosphorus was calculated as the measured serum phosphorus multiplied by 100. As shown in Figure 2, his serum calcium level remained normal, and his serum parathyroid hormone level was initially slightly elevated and subsequently normalized. Serum phosphate levels showed a gradual upward trend, with the most recent measurement of 0.85 mmol/L. Serum creatinine levels fluctuated between 135 μmol/L and 178 μmol/L, with the most recent measurement at 135 μmol/L showing a slight decrease. In recent studies, urine glucose and protein levels have decreased from 3 + to 1+. Table 2 details bone mineral density changes in the patient. The bone density gradually improved and reached normal levels in the most recent assessment. The rib fractures showed signs of healing with callus formation. The symptoms of fatigue and chest pain were resolved. The patient was satisfied with the results of the treatment.

**Table 2:** Bone mineral density follow-up results of the patient.

Date	Bone mineral density (*T*-value)					
	L1 lumbar vertebrae	L2 lumbar vertebrae	L3 lumbar vertebrae	L4 lumbar vertebrae	Femoral neck	Femoral shaft
November 5, 2020	−2.6	−2.2	−2.2	−2.7	/	/
June 24, 2021	−1.4	−0.8	1.0	−0.4	−2.5	−2.1
March 9, 2023	0.3	0.3	1.5	1.5	−1.4	−1.2

## 3. Discussion

This article reports a case of hypophosphatemic osteomalacia caused by the combination of adefovir and tenofovir. The patient initially received adefovir for the treatment. Due to frequent reports of hypophosphatemic osteomalacia caused by adefovir ester,^[2,4]^ doctors in county-level hospitals were alert to the possibility of hypophosphatemia caused by adefovir and switched to tenofovir for the treatment of HBV when low blood phosphate was detected. However, both tenofovir and adefovir ester are nucleoside analogs used for the treatment of HBV, and they can accumulate in the proximal tubules of the kidney, leading to apoptosis of tubular epithelial cells by inhibiting mitochondrial DNA synthesis and reprogramming glucose metabolism. This impairs the reabsorption of phosphate in the proximal convoluted tubules, resulting in excessive phosphate excretion, hypophosphatemia, and renal tubular acidosis, leading to osteomalacia.^[5,6]^ There are few reports of hypophosphatemic osteomalacia caused by tenofovir, which may explain the misdiagnosis and delayed treatment of this patient at the primary hospital. A case of hypophosphatemic osteomalacia in the same patient who sequentially used adefovir and tenofovir was reported by Moon et al,^[7]^ but there are no relevant reports in China.

Laboratory findings typically associated with hypophosphatemic osteomalacia include significantly decreased blood phosphate levels, normal or slightly low blood calcium levels, increased urinary phosphate levels, reduced renal phosphate threshold, elevated alkaline phosphatase levels, and normal or mildly elevated parathyroid hormone levels.^[1]^ In this case, the patient had severe hypophosphatemia and high urine phosphate concentration, but low 24-hour urinary phosphate excretion, suggesting severe hypophosphatemic osteomalacia with a long disease course and depleted endogenous phosphate stores. The patient had mildly elevated parathyroid hormone levels at the time of diagnosis, but normal blood calcium and 25-hydroxyvitamin D levels, indicating secondary elevation of parathyroid hormone due to tenofovir alafenamide medication.^[8]^ Tenofovir alafenamide significantly reduces the synthesis of active vitamin D in the kidneys, leading to decreased functional 25-hydroxyvitamin D levels and suppressed calcium-sensing receptor activity, resulting in secondary parathyroid hormone elevation.^[9,10]^ This is 1 reason why the patient was treated with calcitriol capsules for a long duration. A recent report described secondary hyperparathyroidism caused by tenofovir alafenamide and highlighted that patients with secondary hyperparathyroidism due to tenofovir alafenamide have low-normal levels of serum calcium and 24-hour urinary calcium excretion, distinguishing them from primary hyperparathyroidism.^[11]^ This is inconsistent with the present case. The patient’s symptoms of chest pain and fatigue, along with hypophosphatemia, provided clues to the diagnosis, and whole-body PET-CT confirmed the diagnosis of hypophosphatemic osteomalacia. In rare endocrine disorders, PET-CT can be used to provide valuable diagnostic information. Normal whole-body PET-CT findings could not rule out tumor-induced osteomalacia (TIO). 68Ga-DOTA TATE PET/CT was the first choice followed by OCT PET/CT. After > 2 years of follow-up and routine auxiliary examination, the patient was excluded from a TIO diagnosis. The patient also had glucosuria, proteinuria, renal insufficiency, and hypouricemia, and blood gas analysis suggested metabolic acidosis, leading to a diagnosis of Fanconi syndrome. If conditions and patient consent permit, further diagnostic tests, such as urinary amino acid analysis and renal biopsy, can be performed to confirm the diagnosis. FGF23 plays an important role in phosphate regulation and has clinical significance in the differential diagnosis of hypophosphatemic osteomalacia.^[1,12]^ However, FGF23 testing is not widely available in many hospitals and laboratories, and data were not available in this case report.

For drug-induced hypophosphatemic osteomalacia, the first step was to discontinue medication. In this case, adefovir dipivoxil and tenofovir alafenamide were discontinued, and entecavir disintegration tablets were initiated for antiviral therapy, effectively controlling the patient’s HBV infection. Phosphate supplementation is a basic treatment for hypophosphatemic rickets. In China, there are currently no commercially available phosphate capsules or tablets, and most hospitals use neutral phosphate buffer solutions that need to be prepared on a temporary basis. However, since 2006, many medical institutions have ceased the preparation of compounded formulations in accordance with the relevant regulations, making it difficult to obtain neutral phosphate buffer solutions. During the initial hospitalization, intravenous sodium glycerophosphate was administered. After discharge, the patient was unable to obtain a neutral phosphate oral solution and received phosphate supplementation with sodium fructose diphosphate. However, it was challenging to determine the optimal dosage, and the patient’s blood phosphate levels fluctuated between 0.50 mmol/L and 0.85 mmol/L, remaining below the normal reference range. The latest AACE guidelines for the diagnosis and management of hypophosphatemia suggest that patients with renal Fanconi syndrome should receive treatment with both sodium bicarbonate and phosphate supplementation.^[13]^ In addition to these treatments, the patient followed a high-phosphate diet, received moderate sun exposure, and was prescribed calcitriol capsules for active vitamin D supplementation to improve the bone mass. There have been reports of good clinical efficacy of denosumab in the treatment of hypophosphatemic osteomalacia caused by adefovir dipivoxil, provided that the patient has excessive bone resorption and receives adequate supplementation of vitamin D and phosphate before starting denosumab.^[14]^ Bisphosphonates and RANKL monoclonal antibodies are currently not recommended for the treatment of hypophosphatemic osteomalacia, and require caution.

Hypophosphatemic osteomalacia is relatively rare, and both healthcare professionals and the general public have a limited awareness of this condition. Many patients do not receive timely diagnosis or appropriate treatment. Through a review of the diagnostic and treatment processes in this case, it is evident that there are several practical issues in the diagnosis and treatment of hypophosphatemic osteomalacia, including the limited availability of FGF23 testing and difficulties in obtaining neutral phosphate buffer solutions. In the management of rare diseases, national, provincial, or regional centers for rare diseases should play an active role in conducting unconventional tests and stocking rare drugs to ensure that patients receive timely and appropriate diagnosis, treatment, and comprehensive lifelong medical care, thereby improving their quality of life.

## Acknowledgments

This work was supported by the Zhejiang Traditional Chinese Medicine Science and Technology Plan (2024ZL1286 and 2024ZL206).

## Author contributions

**Conceptualization:** Yinhui He.

**Data curation:** Xin Huang, Yongjun Ye.

**Funding acquisition:** Yinhui He.

**Resources:** Yongjun Ye.

**Software:** Xin Huang, Yongjun Ye.

**Supervision:** Haiyan Xu.

**Visualization:** Xin Huang.

**Writing – original draft:** Yinhui He.

**Writing – review & editing:** Yinhui He, Haiyan Xu.
